# A Structured, Repetitive Simulation Program for Endoscopic Coagulation Hemostasis with Hemostatic Forceps: An Assessor-Blinded Randomized Controlled Trial Using a Bleeding Ulcer Model

**DOI:** 10.1159/dig/acmag013

**Published:** 2026-10-15

**Authors:** Suguo Suzuki, Takeshi Kanno, Yutaka Hatayama, Yutaro Arata, Yohei Ogata, Masahiro Saito, Xiaoyi Jin, Waku Hatta, Kaname Uno, Akira Imatani, Tomoyuki Koike, Atsushi Masamune

**Affiliations:** a Division of Gastroenterology, Tohoku University Graduate School of Medicine, Sendai, Japan; b R and D Division of Career Education for Medical Professionals, Medical Education Center, Jichi Medical University, Shimotsuke, Japan; c Graduate Medical Education Center, Tohoku University Hospital, Sendai, Japan; d Houen-Home Medical Care Support Clinic-Hachiman, Sendai, Japan; e Division of Promotion for Gastroenterological Medical Innovation, Tohoku University, Sendai, Japan

**Keywords:** Hemostasis, Gastrointestinal hemorrhage, Endoscopy, Simulation-based training, Upper gastrointestinal bleeding

## Abstract

**Introduction:**

Prompt endoscopic hemostasis is required to control upper gastrointestinal bleeding. Current training relies on on-the-job exposure, with limited evidence supporting the use of dry-lab simulators. This study aimed to develop a structured, repeatable simulation-based training (SBT) program for thermal coagulation hemostasis and evaluate its efficacy.

**Methods:**

This single-center, assessor-blinded, randomized controlled trial was conducted at Tohoku University Hospital from December 2023 to June 2025. Nonexpert endoscopists were randomized into either an intervention group (five SBT sessions, including basic and hemostasis tasks, over 2 months) or a control group. Co-primary outcomes were procedure time and blinded video-based rubric scores for hemostasis at both low- and high-difficulty sites. Changes in self-efficacy were assessed using a visual analog scale (VAS). The analysis followed intention-to-treat principles.

**Results:**

Twenty participants were enrolled, with nine in the intervention group and 11 in the control group. At the final assessment, the intervention group demonstrated shorter procedure times at both the antrum (43.6 vs. 51.3 s, *p* = 0.02) and the upper gastric body (32.6 vs. 59.7 s, *p* < 0.01). Significant within-group reductions were observed only in the intervention group (effect sizes of 0.62 in the antrum and 0.80 in the upper body). Rubric-based scores improved at high-difficulty sites (*p* = 0.03). All five VAS domains improved significantly in the intervention group, whereas two domains increased in the control group.

**Conclusion:**

Structured SBT with a high-fidelity bleeding simulator improved technical performance, efficiency, and self-efficacy in coagulation hemostasis, representing a scalable framework for competency-based education.

## Introduction

Upper gastrointestinal bleeding (UGIB) is a common and serious emergency condition encountered in gastroenterological practice, arising from the esophagus, stomach, or duodenum, and potentially life-threatening in elderly patients or those with significant comorbidities [1]. Among major etiologies, peptic ulcer bleeding accounts for approximately half of all UGIB cases [2, 3]. Patients with uncontrolled bleeding are at high risk of hemodynamic instability, rebleeding, and poor outcomes [4]. Despite advances in endoscopic and pharmacological management [5], the in-hospital mortality rate of UGIB remains 2–10% [6–9].

Endoscopic hemostasis is recommended as the first-line treatment for UGIB [10, 11]. In our previous study, 70% of the bleeding ulcers were initially managed endoscopically [12]. Endoscopy allows both bleeding source identification and therapeutic interventions, including injection, mechanical methods, thermal coagulation, and topical sprays. Among them, clipping and thermal coagulation are strongly recommended for peptic ulcer bleeding [10]. Success is highly operator dependent [13, 14], and minor errors in manipulation or positioning may result in treatment failure, rebleeding, or adverse events such as perforation.

Currently, skill acquisition depends largely on on-the-job training (OJT). However, in emergency endoscopy, learning opportunities are limited by patient safety concerns and case variability, leading to heterogeneous training quality [15, 16]. In contrast, structured assessment tools have been established for ERCP; the Bethesda ERCP Skill Assessment Tool decomposes procedural steps and provides a rubric-based performance evaluation [17].

Simulation-based training (SBT) has been advocated internationally to address these limitations [18, 19]. SBT enables repetitive practice without patient risk, objective evaluation, and structured feedback [16, 20–22]. Nevertheless, evidence of its effectiveness in endoscopic hemostasis remains limited [23]. Existing simulators include virtual reality (VR), ex vivo animal models, and mechanical models. VR systems enable repeated use and recording of metrics, but they lack realistic bleeding or tissue resistance. Ex vivo models offer realism [24], but raise issues of ethics and infection control. Mechanical simulators are more practical but rarely reproduce active bleeding.

Educational effectiveness is often evaluated using Kirkpatrick’s four-level model, from learner satisfaction (level 1) and knowledge gain (level 2) to behavioral change (level 3) and patient outcomes (level 4) [25]. Most endoscopy education studies stop at levels 1–2, with few assessing whether simulation alters clinical performance (level 3).

We developed a high-fidelity ulcer simulator, “Medical Rising STAR Ulcer-Type,” to address this gap; it can reproduce active bleeding with artificial vessels [24]. A prior study showed the benefits of clip application [16]; however, repetitive training with coagulation forceps, a major modality alongside clipping, has not been systematically evaluated. Therefore, this randomized controlled trial aimed to validate a structured, repetitive SBT program for thermal coagulation hemostasis using this model, with outcomes including objective technical performance and self-efficacy.

## Material and Methods

### Trial Design

This single-center, prospective, parallel-group, assessor-blinded randomized controlled trial evaluated the educational effectiveness of thermal coagulation hemostasis training with hemostatic forceps. The study was conducted between December 2023 and June 2025. The study protocol was approved by the Ethics Committee of the Graduate School of Medicine, Tohoku University on January 23, 2023 (approval number 2022-1-882) and conducted in accordance with the Declaration of Helsinki. This trial was prospectively registered in the University Hospital Medical Information Network (UMIN) Clinical Trials Registry (UMIN000050708 registered on March 30th, 2023). Participants were randomized to either an intervention group, which underwent a structured, repetitive simulation program consisting of five sessions over 2 months, or a control group, which completed assessments only at baseline and at the end of 2 months. The co-primary outcomes were the blinded video-based rubric total scores and time to hemostasis. The primary analysis followed the intention-to-treat principle, with a pre-specified per-protocol sensitivity analysis. Secondary outcomes included changes in subjective evaluations before and after the SBT program in each group.

### Randomization, Concealment of Allocation, and Blinding

Computer randomization was performed using the minimization algorithm in the UMIN INDICE Cloud [26], with a maximum imbalance of four participants between groups. Random seeds were generated that were not accessible to the investigators. Allocation concealment was ensured by using a central web-based system that revealed group assignments only after registration. Investigators, instructors, and assessors had no access to allocation information before enrollment. The participants and instructors were not blinded due to the nature of the intervention; however, the primary outcomes were assessed using anonymized videos evaluated by the same independent expert endoscopist, who was blinded to group allocation and assessment timing.

### Trial Population, Intervention, and Follow-Up

Eligible participants were nonexpert endoscopists at Tohoku University Hospital. Experts were defined as board-certified trainers or endoscopists with ≥10 years of endoscopic experience and were excluded from participation. Accordingly, nonexperts were defined as endoscopists who did not meet either of these criteria and were not considered competent to perform endoscopic hemostasis independently without supervision. Additional exclusion criteria included planned participation in equivalent training during the study period, withdrawal of consent, or safety concerns. The equipment consisted of an upper gastrointestinal lumen model with a bleeding ulcer unit (Medical Rising STAR Ulcer-Type; Denka Co., Ltd., Tokyo, Japan, (online suppl. Fig. 1-a; for all online suppl. material, see https://doi.org/10.1159/dig/acmag013), an endoscope (Q260J; Olympus, Tokyo, Japan), hemostatic forceps (FD-412LR; Olympus, Tokyo, Japan), and an electrosurgical generator (ERBE VIO-3; Swift Coag mode, effect 8.3 standardized). The ulcer unit contained four artificial vessels, with bleeding simulated using red-colored water [24].

All sessions were conducted in accordance with standardized protocols. The intervention group underwent structured sessions consisting of an orientation, two basic tasks (target touching and circumferential hovering), two clinical hemostatic tasks (low difficulty, antral greater curvature; high difficulty, posterior wall of the upper gastric body) (online suppl. Table 1), and rubric-based feedback. The basic clinical tasks were as follows:

Task A: Four 10-mm circles were placed on the anterior wall of the gastric body (online suppl. Fig. 1-b), and trainees touched the center of each circle twice clockwise using forceps.

Task B: Using the same circles, the trainees kept the forceps tip approximately 1 mm from the center of each circle and traced the circumference twice clockwise while maintaining a constant distance.

Task C (low-difficulty hemostasis): Trainees performed hemostasis twice on separate bleeding sites at the antral greater curvature, a location requiring minimal angulation and therefore considered low difficulty (online suppl. Fig. 1-c).

Task D (high-difficulty hemostasis): Trainees performed hemostasis twice on separate bleeding sites at the posterior wall of the upper gastric body, which required retroflexion or downward viewing and was considered highly difficult.

The same anatomical sites were used throughout all training sessions and assessments to ensure standardization and reproducibility. The control group received no training exposure and underwent the same task set at baseline and final assessment. The training schedule itself was designed with reference to previous laparoscopic studies on simulator-based learning, adopting a structure of five sessions over 2 months [27, 28].

### Outcome Measures

Assessments of the primary outcomes were video-recorded at both time points. Hemostasis was defined as successful if achieved within 10 min after bleeding recognition or within five coagulation attempts, including the conversion of pulsatile to oozing bleeding. Secondary outcomes were assessed using a visual analog scale (VAS, 0–100 mm) for the following five domains: (1) *understanding of the procedure*, (2) *confidence in independent hemostasis*, (3) *expectation of simulator effectiveness*, (4) *ability to imagine real clinical procedures*, and (5) *willingness to recommend the simulator*. Analyses were restricted to within-group changes because these measures were subjective self-reports. Adverse clinical events were not applicable in this educational trial, as it did not involve patients. Potential harm related to simulator use (e.g., eye strain, musculoskeletal discomfort, and device malfunction) was monitored, and none was reported.

### Rubric and Assessment

A bilingual (Japanese/English) rubric was developed through discussion between two expert endoscopists with extensive experience in therapeutic endoscopy to objectively assess coagulation hemostasis skills with hemostatic forceps. Each item is scored on a five-point scale from 0 to 4 (0 = not assessable, 1 = poor, 2 = insufficient, 3 = good, and 4 = excellent). A score of three or higher was considered satisfactory, and a score of four was regarded as expert-level performance.

The rubric consisted of six core components and one global assessment:

1. Correct identification of the bleeding site and smooth approach
2. Appropriate distance between the endoscope and the ulcer
3. Stable visual field maintained during the procedure
4. Accurate grasping of the bleeding vessel
5. Adequate coagulation time (neither excessively short nor long)
6. Gentle tissue handling, avoiding excessive traction, pressure, or inappropriate coagulation

The global assessment judged whether the trainee could be entrusted with performing hemostasis independently. The rubric was also used as an educational tool to provide structured feedback aligned with the learning objectives.

### Sample Size Calculations and Statistical Analyses

Sample size was calculated to detect a between-group difference in the co-primary outcomes, assuming an effect size of 1.1, a two-sided α = 0.10, and power = 0.80. Because this study was designed as an exploratory educational trial with a limited number of eligible participants, a less stringent α level was selected to maintain feasibility while reducing the risk of overlooking potentially meaningful educational effects. G*Power 3.1 estimated a total of 16 participants; the target was set at 20 to allow for dropouts. Continuous variables were summarized as medians (interquartile range [IQR]). Between-group comparisons were performed using the Mann-Whitney U test, and within-group comparisons were performed using the Wilcoxon signed-rank test. Nominal variables were analyzed using Fisher’s exact test. The effect sizes for procedure time were reported as Cohen’s d (0.2, 0.5, and 0.8, defined as small, medium, and large, respectively). Statistical significance was set at *p* < 0.05. All statistical analyses were performed using EZR version 1.53 [29].

## Results

### Participant Characteristics

Twenty gastroenterology non-expert doctors at Tohoku University Hospital (17 men and three women) were enrolled between December 2023 and June 2025 after providing oral informed consent. None of the participants was a board-certified specialist at the Japan Gastroenterological Endoscopy Society. Nine participants were allocated to the intervention group and 11 to the control group; there were no dropouts or withdrawals (Fig. 1). Baseline characteristics, including sex distribution, age, postgraduate year, months of endoscopic experience, and number of prior therapeutic endoscopic procedures, did not differ significantly between the groups (Table 1).

**Fig. 1.**
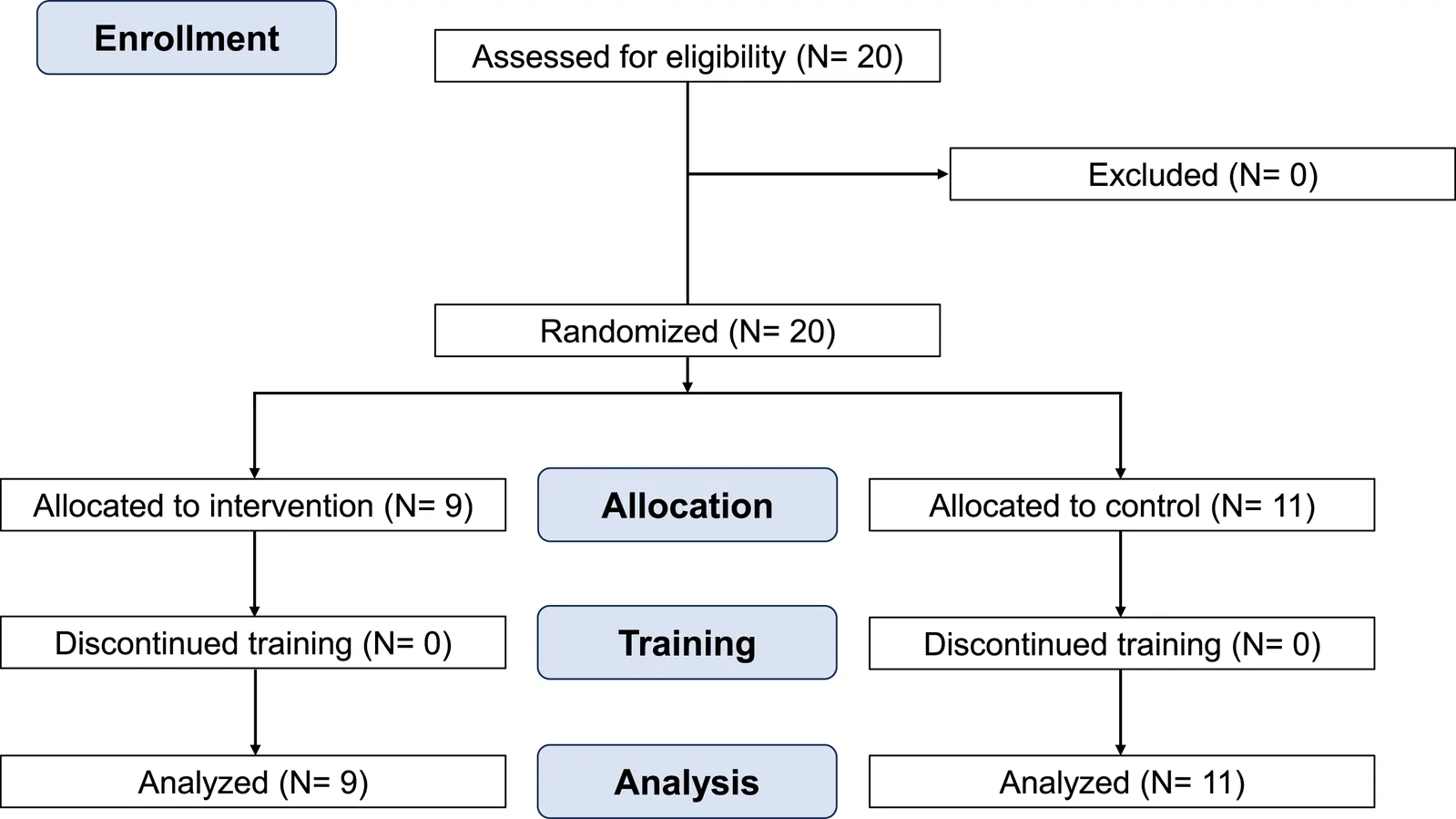
CONSORT participants flowchart. CONSORT flow diagram illustrating enrollment and analysis.

**Table 1.** Participants’ baseline characteristics

	Control group	Intervention group	*p* value
	(*N* = 11)	(*N* = 9)	
Baseline characteristics			
Sex, male/female	9/2	8/1	n.s
Age, years (median [IQR])	31.0 (30.0–32.0)	31.0 (31.0–32.0)	n.s
Postgraduate year, years (median [IQR])	6.0 (6.0–7.0)	6.0 (5.0–7.0)	n.s
Months of endoscopy experience, months (median [IQR])	47.0 (38.5–52.5)	46.0 (35.0–60.0)	n.s
Experience of endoscopic treatment	*Cases(0–50/51-)*		
Endoscopic hemostasis	7/4	5/4	n.s
Hemostatic forceps coagulation	9/2	5/4	n.s
EMR or polypectomy	0/11	0/9	n.s
ESD	9/2	7/2	n.s
ERCP	5/6	5/4	n.s

IQR, interquartile range; EMR, endoscopic mucosal resection; ESD, endoscopic submucosal dissection; ERCP, endoscopic retrograde cholangiopancreatography; postgraduate year, years since graduation from medical school.

### Procedure Time for Hemostasis

At baseline, no significant between-group differences were observed in median procedure time: 55.3 s (IQR 42.2–72.7) in the intervention group vs. 70.5 s (IQR 57.8–95.3) in the control group at the antral greater curvature (low difficulty, *p* = 0.07) and 74.7 s (IQR 47.0–90.7) vs. 66.1 s (IQR 41.0–82.0) at the upper gastric body (high-difficulty, *p* = 0.60) (Fig. 2). At the final assessment, procedure times were significantly shorter in the intervention group at both sites: 43.6 s (IQR 32.0–46.2) vs. 51.3 s (IQR 38.6–95.5) at the antrum (*p* = 0.02) and 32.6 s (IQR 24.4–46.4) vs. 59.7 s (IQR 50.5–72.1) at the upper gastric body (*p* < 0.01). No hemostasis failure was observed in any assessment.

**Fig. 2.**
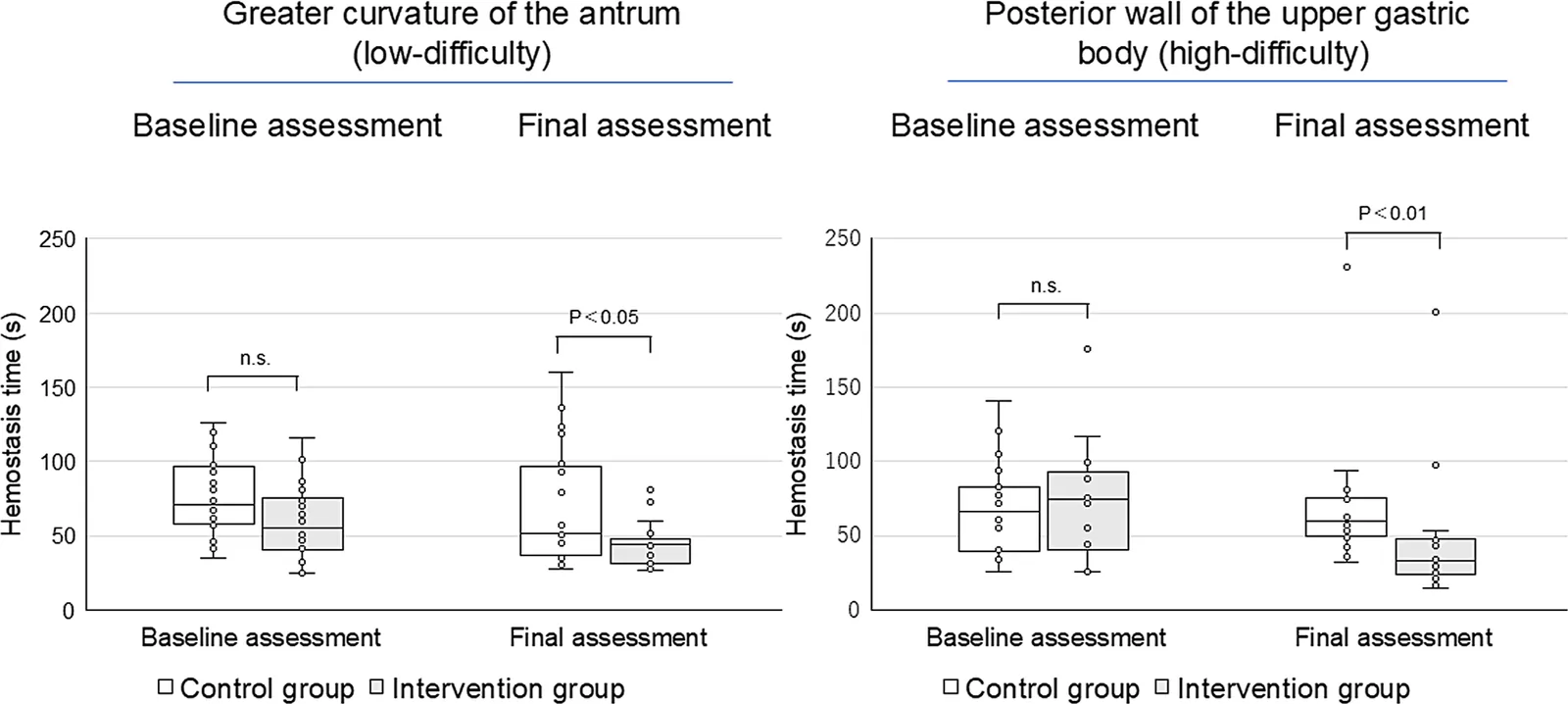
Comparison of hemostasis procedure times between the intervention and control groups at baseline and final assessments. Data are presented as medians with interquartile ranges, and *p* values were calculated using the Mann-Whitney U test.

In the intervention group, median procedure times decreased significantly from 55.3 s (IQR 42.2–72.7) to 43.6 s (IQR 32.0–46.2) at the antrum (*p* = 0.03, effect size 0.62) and from 74.7 s (IQR 47.0–90.7) to 32.6 s (IQR 24.4–46.3) at the upper gastric body (*p* < 0.01, effect size 0.80). In the control group, no significant changes were observed at any of the sites (Fig. 3).

**Fig. 3.**
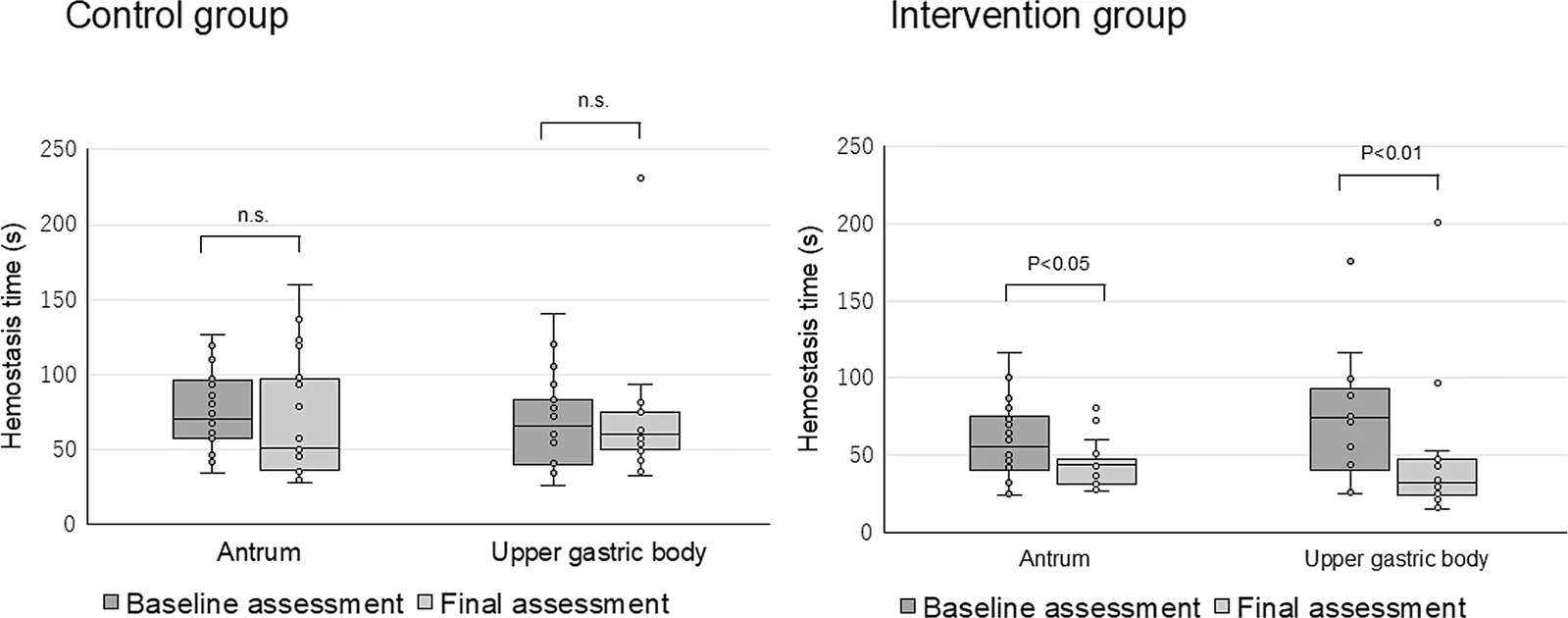
Comparison of hemostasis procedure times between baseline and final assessments within each group. Data are presented as medians with interquartile ranges, and *p* values were calculated using the Wilcoxon signed rank test.

### Blinded Video Evaluation of Technical Elements according to Rubrics

Six technical elements and a global assessment were scored on a 0–4 scale (maximum 28 points). At baseline, no significant differences were observed in median scores: 20.0 points (IQR 18.0–21.0) in the intervention group vs. 18.0 points (IQR 16.0–20.5) in the control group at the antrum (*p* = 0.30), and 16.0 points (IQR 16.0–17.0) vs. 16.0 points (IQR 14.5–17.0) at the upper gastric body (*p* = 0.82). At the final assessment, no difference was seen at the antrum (21.0 points (IQR 20.0–22.0) vs. 18.0 points (IQR 18.0–20.5), *p* = 0.17), but the intervention group tended to achieve higher scores at the upper gastric body (20.0 points (IQR 18.0–22.0) vs. 17.0 points (IQR 16.5–18.5), *p* = 0.06). From baseline to final assessment, the intervention group demonstrated a significant increase in rubric scores at the high-difficulty site (upper gastric body: 16.0 points (IQR 16.0–17.0) at baseline vs. 20.0 points (IQR 18.0–22.0) at final assessment, *p* = 0.03), while no significant change was observed at the low-difficulty antrum (*p* = 0.18). The control group showed no significant changes in scores at either site (Fig. 4). The detailed scores for each rubric item are provided in online supplementary Table 2.

**Fig. 4.**
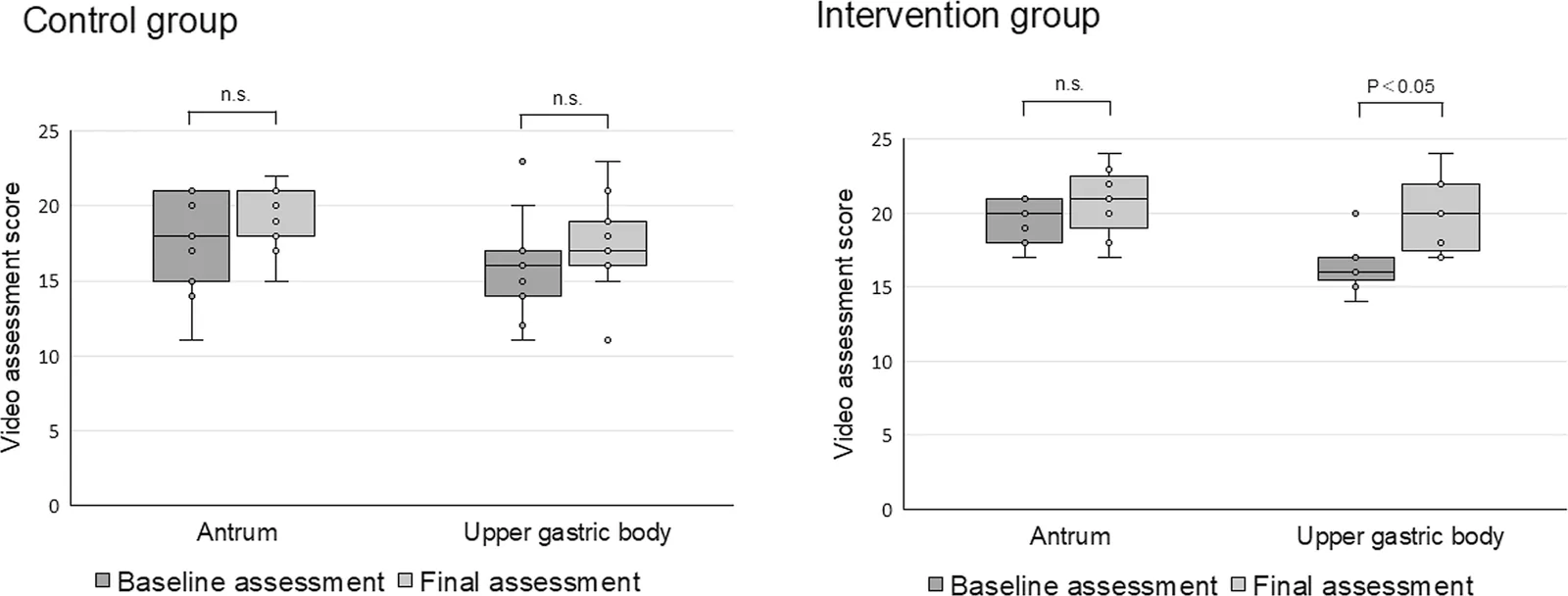
Comparison of video evaluation scores between baseline and final assessments within each group. Data are presented as medians with interquartile ranges, and *p* values were calculated using the Wilcoxon signed-rank test. Detailed item-level scores are provided in online supplementary Table 2.

### Subjective Evaluation Using VAS Scores

From baseline to final assessment (Table 2), the intervention group demonstrated significant improvement across all five domains: understanding of the procedure increased from 74.0 (IQR 55.0–75.0) to 86.0 (IQR 86.0–90.0, *p* < 0.01); confidence in independent hemostasis from 62.0 (IQR 49.0–74.0) to 87.0 (IQR 83.0–92.0, *p* < 0.01); expectation of simulator effectiveness from 81.0 (IQR 73.0–82.0) to 91.0 (IQR 89.0–97.0, *p* < 0.05); ability to imagine real clinical procedures from 74.0 (IQR 63.0–77.0) to 91.0 (IQR 90.0–96.0, *p* < 0.01); and willingness to recommend the simulator from 90.0 (IQR 73.0–96.0) to 91.0 (IQR 90.0–95.0, *p* < 0.05). In the control group, improvements were observed in understanding of the procedure (63.0 (IQR 55.0–69.0) to 79.0 (IQR 69.0–84.0), *p* < 0.01) and confidence in independent hemostasis (49.0 (IQR 43.5–60.5) to 67.0 (IQR 59.5–69.0), *p* < 0.05). No significant changes were detected in the expectation of simulator effectiveness, ability to imagine real clinical procedures, or willingness to recommend the simulator.

**Table 2.** Comparison of VAS scores between baseline and final assessments within each group

	Baseline questionnaire	Final questionnaire	*p* value
	VAS, median (IQR)		
Control group			
Q1 Understanding of the procedure	63.0 (55.0–69.0)	79.0 (69.0–84.0)	**<0.01**
Q2 Confidence in performing hemostasis independently	49.0 (43.5–60.5)	67.0 (59.5–69.0)	**<0.01**
Q3 Perception of the simulator’s effectiveness for skill improvement	74.0 (68.5–78.0)	83.0 (78.0–88.0)	n.s.(0.06)
Q4 Ability to imagine real clinical procedures during training	82.0 (74.5–87.5)	79.0 (73.0–85.5)	n.s
Q5 Willingness to recommend the simulator to colleagues	83.0 (81.0–92.0)	83.0 (76.0–91.0)	n.s
Intervention group			
Q1 Understanding of the procedure	74.0 (55.0–75.0)	86.0 (86.0–90.0)	**<0.01**
Q2 Confidence in performing hemostasis independently	62.0 (49.0–74.0)	87.0 (83.0–92.0)	**<0.01**
Q3 Perception of the simulator’s effectiveness for skill improvement	81.0 (73.0–82.0)	91.0 (89.0–97.0)	**<0.01**
Q4 Ability to imagine real clinical procedures during training	74.0 (63.0–77.0)	91.0 (90.0–96.0)	**<0.01**
Q5 Willingness to recommend the simulator to colleagues	90.0 (73.0–96.0)	91.0 (90.0–95.0)	**0.04**

IQR, interquartile range; VAS, visual analogue scale. Bold values indicate statistical significance (*p* < 0.05).

## Discussion

This randomized controlled trial demonstrated that a structured, repetitive training program for thermal coagulation hemostasis significantly improved the performance of early-career gastroenterologists. The intervention group achieved shorter procedure times at both low- and high-difficulty sites, with significant within-group reductions and large effect sizes observed only in this group. Rubric-based video scores improved at the high-difficulty site, indicating that the program resulted in measurable technical enhancement consistent with Level 3 of the Kirkpatrick model [25].

Traditionally, training in endoscopic hemostasis has relied mainly on OJT. Although OJT provides valuable real-world exposure, its opportunities are limited and uneven, particularly in emergency settings, highlighting the need for alternative educational approaches [15, 16]. Simulation-based education has become a standard component of training in other surgical fields. For example, the completion of validated programs such as the Fundamentals of Laparoscopic Surgery is required for board certification in the USA [30], and strong evidence supports their efficacy in improving operative performance and autonomy [31]. Compared with this well-established framework, gastroenterology lags behind in the systematic adoption of simulation and validated assessment methods [32]. Our findings suggest that structured SBT can address this gap in endoscopic hemostasis training.

Previous studies have primarily evaluated hemostasis training using ex vivo animal models, such as the compactEASIE system. For example, Maiss et al. reported short-term gains after a single-day compactEASIE course in a pre-post design [33]; Hochberger et al. demonstrated significant improvements with repeated compactEASIE workshops in an RCT [23]; and Haycock et al. confirmed improved performance metrics in another ex vivo randomized comparison [34]. Collectively, these studies confirm that ex vivo training can accelerate skill acquisition. However, ex vivo models present several challenges, including ethical considerations, infection control requirements, and the logistical need for dedicated endoscopes and equipment separate from those used for humans, which limits their feasibility in many hospitals. In contrast, the dry-lab simulator used in the present study reproduces active bleeding without animal tissue [24], is compatible with standard endoscopic equipment, and can be reused with minimal maintenance. These features make it a practical and scalable platform for standardized hemostasis training.

Procedure time, an objective indicator of technical performance, was significantly reduced in the intervention group, particularly at the high-difficulty site in the posterior wall of the upper gastric body. This supports prior evidence that SBT is particularly effective for tasks that require technical expertise [32]. Although all participants achieved successful hemostasis within the predefined simulator-based criteria, shorter procedure times remain an educationally meaningful performance metric and may be relevant in clinical contexts because more efficient bleeding control could reduce ongoing blood loss and facilitate subsequent therapeutic interventions. The 10-min criterion was a simulator-based threshold for task completion and should not be viewed as a clinically acceptable duration for unsupervised trainee management of spurting bleeding. Nevertheless, the observed procedure times may provide preliminary benchmarks for supervised hemostasis training and instructor intervention. Correspondingly, rubric-based scores improved at the high-difficulty site, whereas no significant improvement was observed at the low-difficulty antral site, likely reflecting a ceiling effect in participants with baseline endoscopic experience rather than complete novices. Thus, the rubric framework appears sensitive to differentiating performance gains under challenging anatomical conditions. Participants had previous exposure to therapeutic endoscopy but had not yet achieved expert-level competency, reflecting the target population that commonly performs hemostatic procedures under supervision in our clinical training setting. The observed improvements may be better understood as enhanced integrated simulator-based hemostatic performance rather than acquisition of a single isolated skill. The program decomposed the goal of hemostasis into structured rubric domains and provided repeated opportunities to identify and refine technical weaknesses in a realistic bleeding environment, integrating basic point-directed and surface-oriented forceps control with hemostasis-specific performance requirements. Subjective outcomes aligned with these findings, as all VAS domains improved in the intervention group. Modest improvements in understanding and confidence in the control group may reflect repeated exposure to the simulator; however, self-reported measures should be interpreted cautiously.

Rubric-based assessment using equal item weighting, consistent with established tools such as the Bethesda ERCP Skill Assessment Tool [17], effectively detected performance gains at high-difficulty sites. While alternative weighting or level-adjusted rubrics may enhance sensitivity, the current framework appears sufficient for facilitating structured learning among early-career gastroenterologists. This framework can be adapted for other therapeutic endoscopic procedures, such as clipping, EMR, ESD, ERCP, and endoscopic ultrasound-guided interventions. Integration with digital technologies, including VR/augmented reality simulators and remote feedback platforms, can extend access to training opportunities across diverse institutions and healthcare systems.

This study has several limitations. While our goal is to improve patient outcomes (e.g., successful hemostasis and reduced blood loss through decreased procedure time), the direct impact of our educational program on these clinical outcomes remains unknown, as the effects were assessed exclusively in a simulation setting. While the simulator reproduces active bleeding and key technical aspects of endoscopic hemostasis, it does not fully replicate the complexity of actual clinical bleeding situations, including ulcer fibrosis, clot formation, peristalsis, respiratory movement, patient instability, and medication- or comorbidity-related bleeding tendency. Conducting clinical RCTs in UGIB is inherently challenging due to case heterogeneity and the emergency nature of the condition [35]. This makes it difficult to select a homogeneous cohort for standardized technical evaluation. Consequently, the simulator provides a standardized and feasible platform for initial competency assessment and is a necessary first step toward competency-based training. Additionally, this study was conducted at a single institution with a small sample size, which may limit the generalizability of our findings. Furthermore, exposure to actual gastrointestinal bleeding cases during the study period was not systematically recorded and therefore could not be controlled for in the analysis. Although participants were randomized, differences in such clinical exposure may have influenced performance independently of the simulation program. All video assessments were performed by a single blinded expert evaluator, which helped ensure consistency across assessments but precluded evaluation of inter-rater reliability. Future studies should evaluate the reliability and validity of the rubric using multiple independent assessors. In addition, no hemostasis failures occurred during the assessments, which may have limited our ability to evaluate failure-related outcomes or discriminate performance at the lower end of the proficiency spectrum. Because this study was exploratory, no formal adjustment for multiplicity was applied; therefore, secondary and exploratory outcomes should be interpreted with caution. Future studies should validate the relationship between simulator-based performance metrics and long-term clinical outcomes in larger multicenter cohorts.

## Conclusion

This randomized controlled trial demonstrated that structured training with a high-fidelity dry-lab simulator significantly improved procedure time, technical performance, and self-efficacy in coagulation hemostasis. This program provides a reproducible and scalable framework for standardized training, with the potential to advance competency-based education in therapeutic endoscopy.

## Statement of Ethics

Verbal informed consent was obtained from all participants prior to the study. The study protocol and this consent procedure and was reviewed and approved by the Ethics Committee of the Graduate School of Medicine, Tohoku University, approval no.: 2022-1-882, decision date January 23, 2023.

## Conflict of Interest Statement

Dr. Takeshi Kanno has a joint research fund from Denka Co., Ltd. Denka Co., Ltd. provided the simulator used in this study and supported the research through a collaborative research fund. However, the company had no role in the study design, data collection, data analysis, interpretation of the data, manuscript preparation, or the decision to submit the manuscript for publication. The other authors declare that they have no conflicts of interest. Dr. Waku Hatta was a member of the journal’s Editorial Board at the time of submission.

## Funding Sources

This study was funded by the Japan Society for the Promotion of Science (Grant No.: 22K10460 [roles: study design, execution, and analysis], 26K03047 [roles: manuscript conception and English writing]).

## Author Contributions

Conception and design (S.S., T.Kanno, and Y.A.); analysis, interpretation of the data, and drafting of the article (S.S. and T.Kanno); critical revision of the article for important intellectual content (Y.H., Y.O., M.S., X.J., W.H., K.U., A.I., and T. Koike); final approval of the article (A.M.)

## Data Availability

The data that support the findings of this study are not publicly available due to privacy reasons but are available from the corresponding author upon request.
